# Dorsal Scapular Osteochondroma in the Chiropractic Clinic: A Case Report and Literature Review

**DOI:** 10.7759/cureus.42007

**Published:** 2023-07-17

**Authors:** Eric Chun-Pu Chu, Edouard Sabourdy

**Affiliations:** 1 Chiropractic and Physiotherapy Centre, New York Medical Group, Hong Kong, CHN; 2 Chiropractic Clinic, FV (Franco-Vietnamese) Hospital, Ho Chi Minh City, VNM

**Keywords:** chiropractor, chiropractic, scapular tumor, osteochondroma, dorsal scapula

## Abstract

Osteochondromas are benign bone tumors that occur only rarely in the scapula. We report a case of an eight-year-old boy with dorsal scapular osteochondroma that initially presented as middle back pain, scoliosis, and a winged scapula. Unlike in most reported cases, the patient did not report severe pain. The chiropractor reviewed his previous radiograph and ordered a computed tomography scan, which revealed a bony mass on the dorsal aspect of the left scapula consistent with an osteochondroma. Given the patient's age and lack of symptoms, a conservative chiropractic treatment plan, focusing on posture, flexibility, and close monitoring, was adopted. Scapular mobilization, spinal adjustment, and a tailored exercise program provided symptomatic relief from scoliosis and improved mobility. Follow-up evaluations revealed further enlargement of the osteochondroma without accompanying symptoms. This study highlights the importance of considering a broad range of differential diagnoses in pediatric patients with atypical or unclear presentations. This study also demonstrates the potential benefits of conservative chiropractic management in patients with osteochondroma-induced musculoskeletal issues.

## Introduction

Osteochondromas are benign bone tumors originating from growth plates that predominantly occur in long bones [1]. Although they account for 4% of all tumors occurring in the scapula, making them the most common tumor type at this location [2], they mostly occur in individuals under 20 years of age, as tumor growth is typically prevented by closure of the physis [2]. Osteochondromas tend to be identified incidentally, as they are often asymptomatic [2]. However, some cases may present with symptoms stemming from osseous deformities, fractures, bursa formation, or mechanical compression of adjacent structures [2]. Very rarely, malignant transformation can occur, particularly in patients with hereditary multiple exostosis [2]. In cases of pain, cosmetic concerns, complications, a high risk of malignant transformation, or an uncertain diagnosis, surgery is indicated [2]. The majority of reported cases of osteochondroma have been located in the ventral scapula [2], with only 10 reported cases of osteochondroma in the dorsal scapula [2-10].

Here, we present the novel case of an eight-year-old boy who presented at a chiropractic clinic with chief complaints of middle back pain and scoliosis and was subsequently diagnosed with dorsal scapular osteochondroma. In contrast to all previously reported cases of osteochondroma in the dorsal scapula [2-10], this case report is the first to demonstrate the benefits of a conservative management approach for the treatment of symptoms related to dorsal scapular osteochondroma, as the majority of previous reports have involved surgery.

## Case presentation

An eight-year-old boy was brought to a chiropractic clinic by his parents with chief complaints of middle back pain, uneven shoulders, a winged scapula, and scoliosis. His medical history revealed that he had been examined by a chiropractor six months previously and was undergoing treatment for flat feet. Radiography was performed to determine the cause of the winged scapula, but the findings were considered unremarkable. The patient was in good general health, with no reported pain, systemic disease, or family history of scoliosis. The patient's mother reported that he was active in school and participated in sports but occasionally complained of discomfort in his left shoulder area after physical activity.

**Figure 1:**
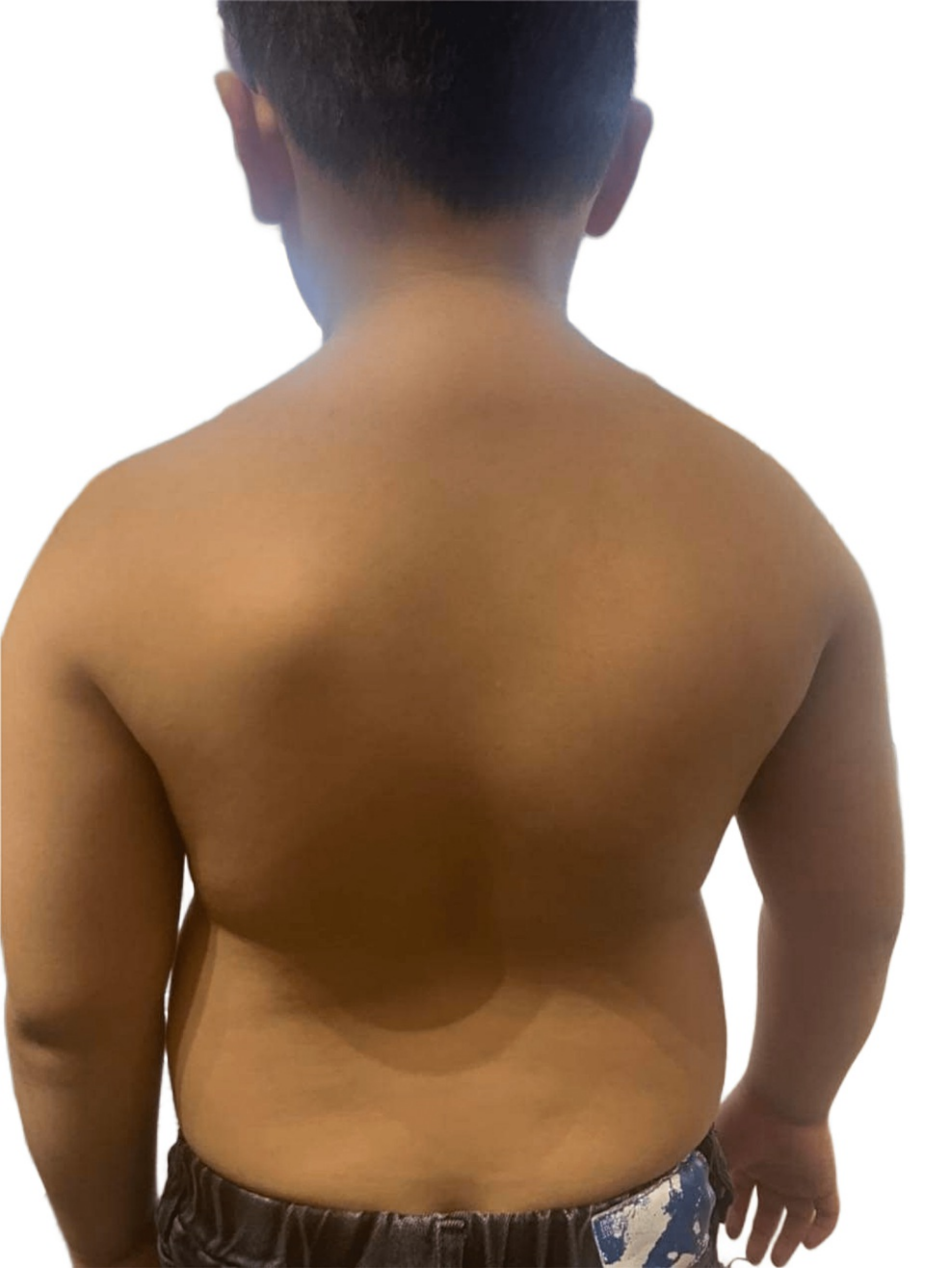
Photo of the back of the patient Upon initial observation, the patient exhibited normal shoulder balance but had a noticeably protruding left scapula.

**Figure 2:**
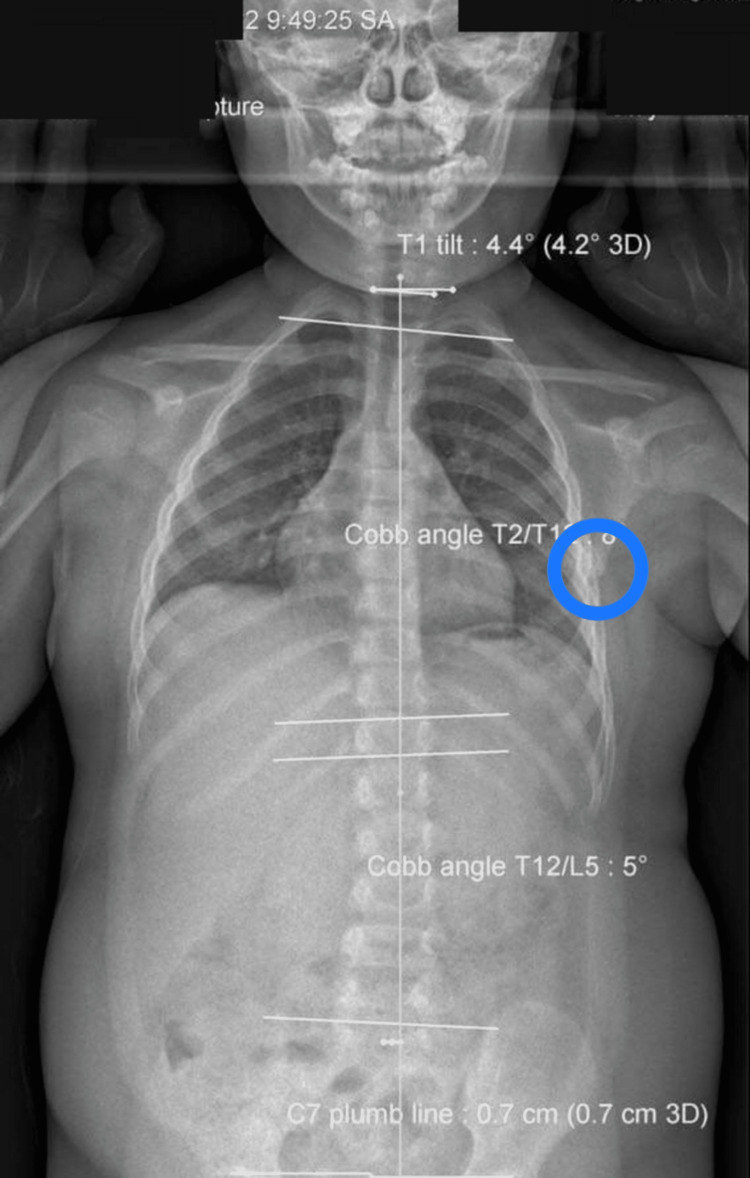
Frontal full-body radiograph A review of X-rays performed six months previously showed minimal signs of thoracic deviation (8° Cobb angle T1-T12, apex T6-7 right). A high-density mass was identified at the inferior angle of the scapula (blue circle).

Initial observation of the patient revealed normal shoulder balance but a noticeably protruding left scapula (Figure 1). A review of the radiographs performed six months previously showed minimal signs of thoracic deviation (8° Cobb angle T1-T12, apex T6-7 right) (Figure 2). The patient was next assessed in both the standing and seated positions. In the standing position, mild elevation of the right shoulder was observed along with increased convexity on the right side of the thoracic spine. In the seated position, the patient tended to lean to the right, possibly to compensate for the discomfort caused by the protruding left scapula. Although the cervical and lumbar ranges of motion were within normal limits, the patient exhibited a decreased range of motion in thoracic lateral flexion and rotation, particularly toward the left side. A hard, immobile mass, which was tender upon palpation, was detected on the left scapula. Mild tenderness and muscle tightness were observed in the left paraspinal muscles of the thoracic spine. The patient’s muscle strength, reflexes, and sensory test results were normal, although Adam's forward bend test revealed a mild rib hump on the left side. The results of the Spurling and straight leg raise tests were unremarkable. No significant leg-length discrepancy was observed with the patient in the supine position, suggesting that scoliosis was not secondary to a functional leg-length inequality.

**Figure 3:**
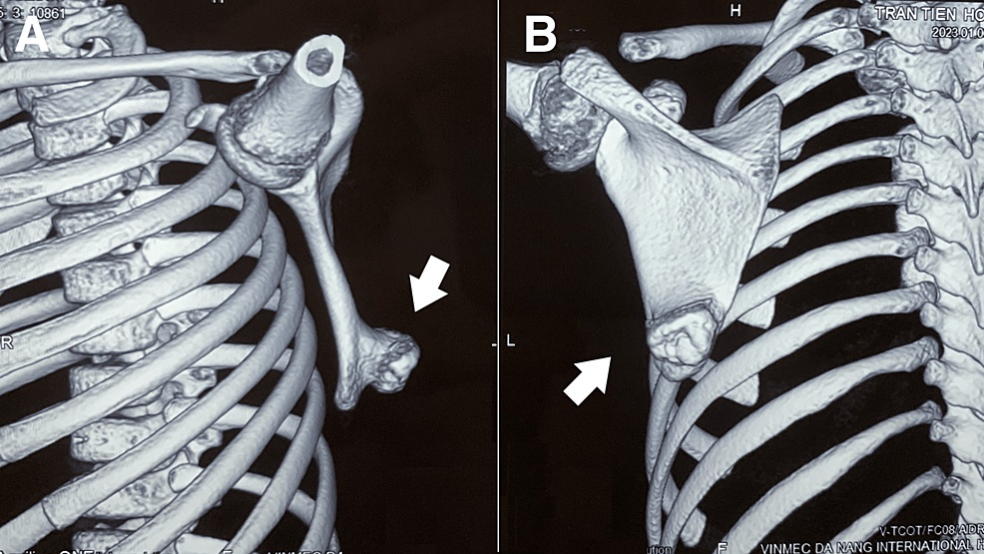
CT scan of the left scapula An osteochondroma can be seen on the dorsal aspect of the left scapula (white arrow) in the (A) lateral and (B) dorsal views.

Suspecting an exostosis, the chiropractor ordered a computed tomography (CT) scan for further investigation. CT confirmed the presence of an exostosis, specifically an osteochondroma, measuring 2.3 x 1.3 cm, on the dorsal aspect of the scapula (Figure 3). Consequently, the patient was diagnosed with osteochondroma. Given the patient's age, a conservative management approach was adopted.

**Figure 4:**
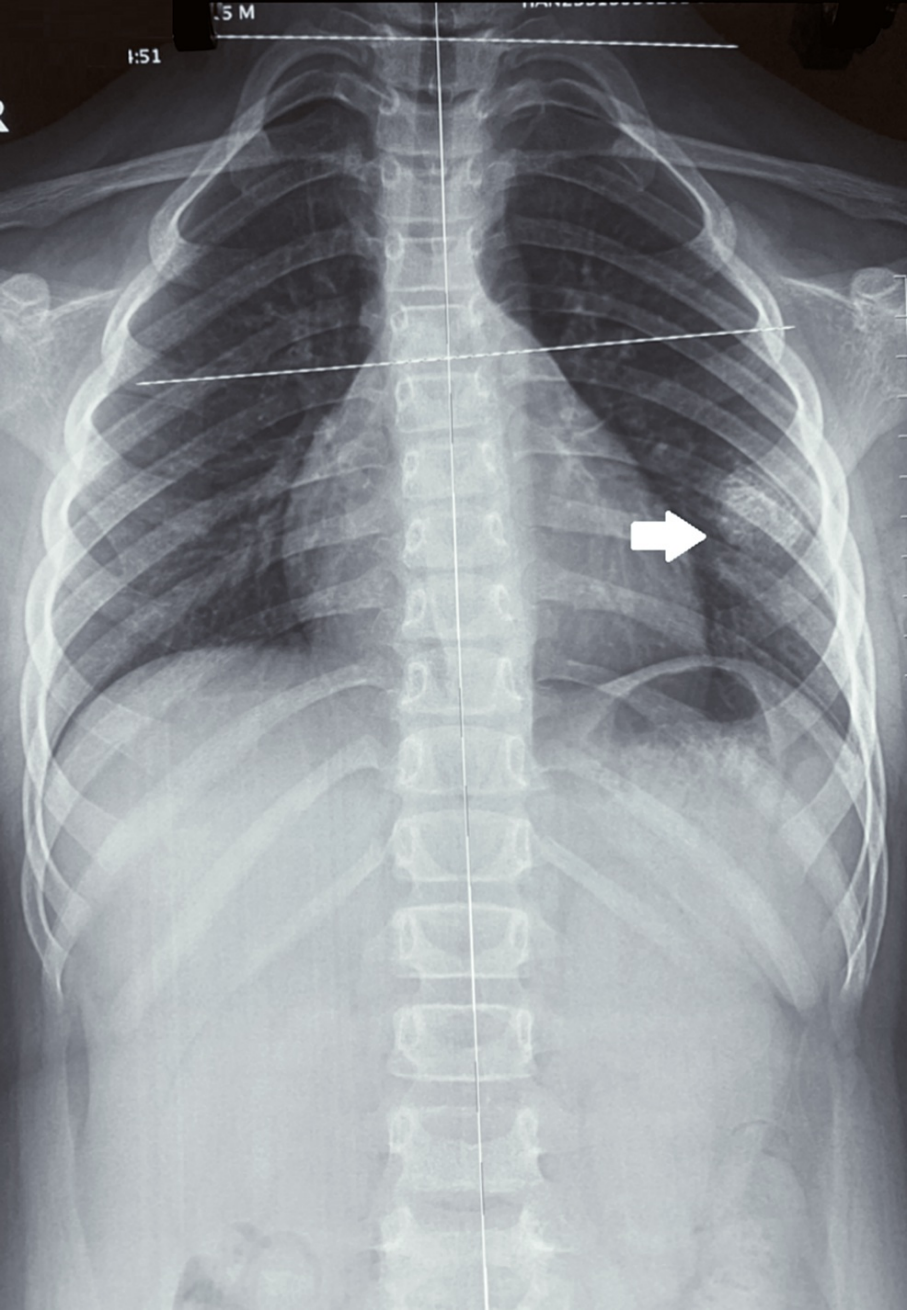
Follow-up radiograph Radiograph performed six months after the initial presentation showed that the mass had grown in size (white arrow).

**Figure 5:**
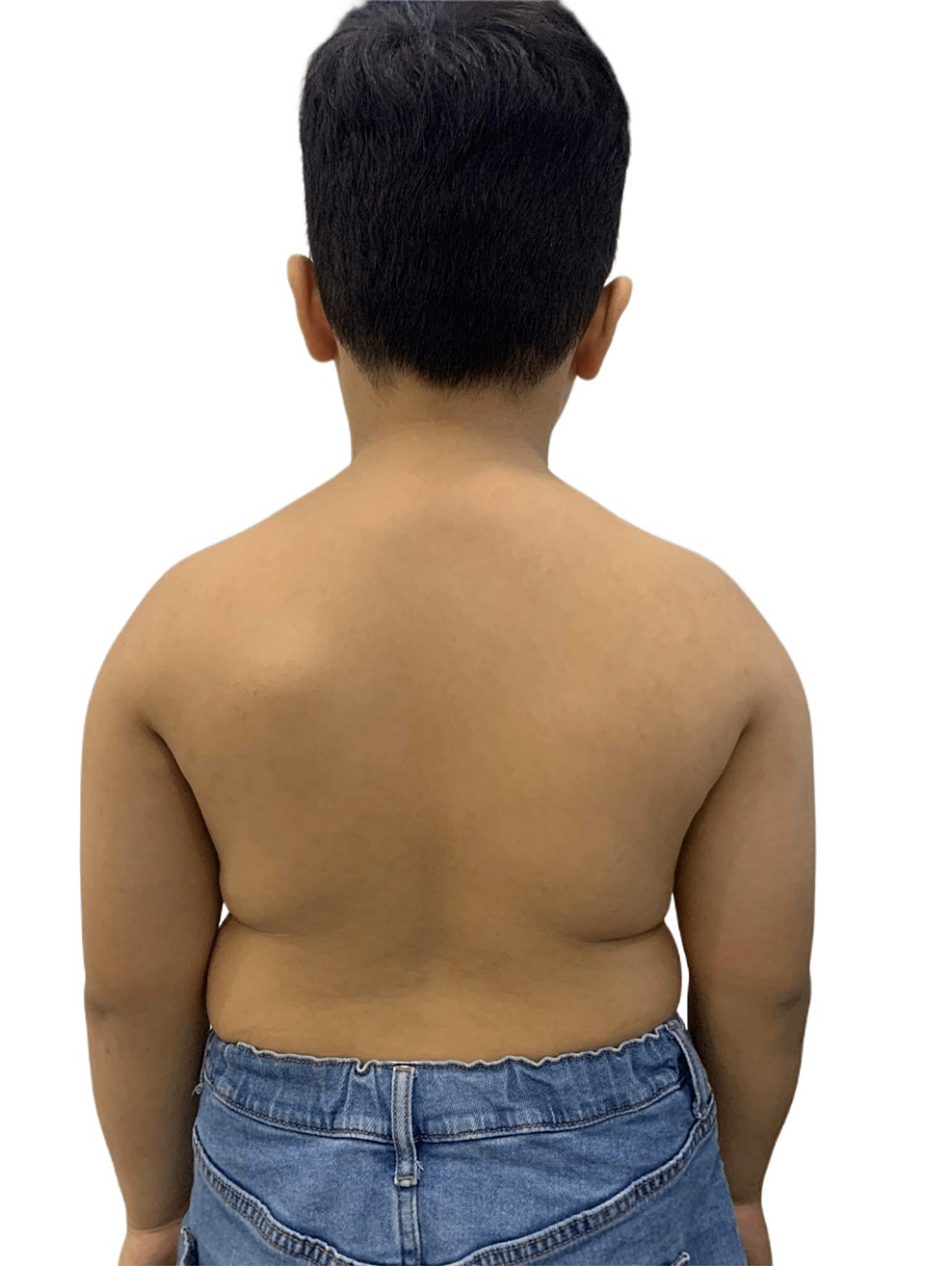
Follow-up photo of the back Photography performed six months after the initial presentation showed a similar appearance of the left scapula.

The chiropractic treatment plan focused on addressing the patient's posture, maintaining flexibility and strength, and monitoring the patient and the osteochondroma for any signs of functional impairment or compression syndrome. The chiropractor performed scapular mobilization and spinal adjustments to improve joint mobility and alignment, particularly in the thoracic spine. This was aimed at alleviating muscle tension and improving overall spinal function. The patient was prescribed a tailored exercise program focusing on stretching the tight muscles and strengthening the weak muscles associated with the scapula. The patient was scheduled for follow-up appointments every six months to monitor the progression of scoliosis and the osteochondroma. At each visit, the chiropractor evaluated the patient's posture, range of motion, and shoulder pain, adjusting the treatment plan as needed. Progress was discussed with the orthopedic surgeon, ensuring a collaborative approach to patient care. By implementing a comprehensive, conservative chiropractic treatment plan, the patient’s postural issues improved. His range of motion was restored at the six-month follow-up. Radiography (Figure 4) and photography (Figure 5) performed at this visit revealed the growth of the mass; however, no new symptoms were associated with this growth. The conservative management approach adopted in this case aimed to optimize the spinal health and overall well-being of the patient to support his continued growth toward adulthood.

## Discussion

Following a search of the PubMed, Scopus, and Google Scholar databases, a total of 10 previous case reports of dorsal scapular osteochondroma were identified; including this case, these cases are summarized in Table 1. The average age of patients in these cases was 10 years; 72% of patients were male. Our case involved an eight-year-old male with a distinctive presentation of scoliosis without shoulder pain; 27% of patients in previously reported cases experienced worsening pain [3,8,9]. In 27% of previous cases, patients reported difficulty sleeping in the supine position [3,5,8], which was not a factor in our case. Our patient also had mild restriction of the scapula, similar to 9% of previous cases that experienced a limited range of motion in their joints [4].

**Table 1:** Case reports of dorsal scapular osteochondromas

Author (year)	Sex	Age, years	Presentation	Location	Treatment	Histology diagnosis	Tumor size, cm	Follow-up
Altwaijri et al. (2022) [2]	F	2	Swelling that worsens and a painful mass	Dorsal	Complete osteochondroma excision	Yes	3.0 x 2.5 cm	Positive after 6 months
Yadkikar et al. (2013) [3]	F	11	Gradually progressive pain and inability to sleep in the supine position	Dorsal	Excisional biopsy	Yes	3.0 x 2.0 cm	Positive after 12 months
Kumar et al. (2014) [4]	M	4	Progressive swelling and restricted movement	Dorsal	Complete osteochondroma excision	Yes	3.0 x 1.7 cm	Positive after 6 months
Jadhav et al. (2016) [5]	M	12	Difficulty in sleeping in the supine position	Dorsal	Excised en mass	Yes	4.0 x 3.0 cm	Positive after 12 months
Nekkanti et al. (2018) [6] (Case 1)	M	19	In the supine position, pain and swelling get worse over time	Dorsal	Complete osteochondroma excision	Yes	3.0 x 3.0 cm	NA
Nekkanti et al. (2018) [6] (Case 2)	M	5	Swelling that gets worse over time	Dorsal	Complete osteochondroma excision	Yes	1.5 x 1.0 cm	NA
Matthewson et al. (2019) [7]	M	2	Having a non-painful mass in his right shoulder	Dorsal	Complete osteochondroma excision	Yes	8.4 x 7.2 x 10.1 cm	NA
Bektas et al. (2019) [8]	F	15	A lump on the left side of the upper back, the inability to sleep on the back, painful shoulder movement, and cosmetic discomfort	Dorsal	An osteotome was used to remove the mass	Yes	NA	Positive after 6 months
Sánchez et al. (2021) [9]	M	11	Painful mass	Inferior- dorsal	The tumor mass was accessed and resected en bloc after the teres minor muscle was only partially detached	Yes	4.0 x 2.8 cm	Positive after 6 months
Shahid et al. (2021) [10]	M	23	Painless longstanding protrusion over the scapula	Dorsal	Observation	NA	NA	NA
Chu et al. (2023)	M	8	Mid back pain and scoliosis	Dorsal	Conservative management of symptoms	NA	2.3 x 1.3 cm	Positive after 6 months

Chiropractors often encounter patients who present with back pain and scoliosis [11-14]. While a winged scapula and scoliosis were the initial concerns in the present case, further investigation revealed a dorsal scapular osteochondroma to be the primary cause of the patient's symptoms. By maintaining a high degree of clinical suspicion and performing thorough investigations, osteochondroma can be diagnosed through advanced radiography, although the diagnosis can also be confirmed histologically during surgery [2]. The treating chiropractor can therefore identify the underlying cause of the patient's symptoms and devise an appropriate management plan [15]. This case serves as a valuable reminder for healthcare practitioners to remain vigilant in their assessments and to consider a broad range of differential diagnoses, especially in pediatric patients with atypical presentations.

In the present case, conservative chiropractic management improved the osteochondroma-induced musculoskeletal symptoms. In the absence of precise evidence-based guidelines, pain management in children with musculoskeletal injuries remains insufficient and highly variable [16]. By focusing on scapular mobilization and spinal adjustments, the chiropractor addressed mobility issues, reduced musculoskeletal pain, improved spinal function, and maintained flexibility. This non-invasive approach minimizes the risk of complications associated with surgical intervention. Moreover, the close monitoring of the patient and the osteochondroma progression through scheduled follow-up appointments ensured that any changes in the patient's condition could be responded to promptly.

The role of chiropractic care in managing pediatric musculoskeletal conditions is multifaceted and extends beyond the provision of manual therapy [17-19]. Chiropractors play a crucial role in patient education by offering guidance on appropriate exercises, postural habits, and lifestyle modifications to support optimal musculoskeletal health [20]. Additionally, chiropractors can help identify and address any biomechanical imbalance or functional deficit that may contribute to the development or exacerbation of musculoskeletal issues [21]. In the present case, the chiropractor's comprehensive approach not only addressed the patient's immediate symptoms but also aimed to optimize his overall spinal health and well-being throughout his growth and development.

## Conclusions

The current study describes a rare presentation of an osteochondroma in an eight-year-old boy, which manifested as scoliosis and a winged scapula. The chiropractor maintained a high index of suspicion and ordered advanced imaging, which led to a diagnosis of dorsal scapular osteochondroma. Unlike the surgical management of previously reported cases, the patient, in this case, benefited from conservative chiropractic care focused on posture, flexibility, and close monitoring. Regular chiropractic adjustments and a tailored home exercise program provided effective relief from symptoms and improved mobility. This case highlights the importance of considering a broad range of differential diagnoses in pediatric patients with musculoskeletal complaints and demonstrates the benefits of conservative chiropractic management in patients with osteochondroma.
